# Double-Sided Illumination Grating-Coupled Surface Plasmon Resonance Sensors Using Direct Optical Discs

**DOI:** 10.3390/ma19030603

**Published:** 2026-02-04

**Authors:** Wisansaya Jaikeandee, Asad Ullah Hil Gulib, Taeyul Choi, Richard Z. Zhang

**Affiliations:** Department of Mechanical Engineering, University of North Texas, Denton, TX 76207, USA; wisansaya.jaikeandee@unt.edu (W.J.);

**Keywords:** surface plasmon resonance, optical disc, grating-coupled plasmonic sensor, SP dispersion

## Abstract

**Highlights:**

**What are the main findings?**

- Compared BD-R, DVD-R, and CD-R as low-cost grating substrates for GC-SPR.
- Identified SP dispersion modes of each optical disc consistent with SPR resonances.
- Front versus back illumination changes resonance clarity and spectrum shape.
- Demonstrated Ag and Cu as plasmonic materials for low-cost GC-SPR platforms.
- RI sensitivity depends on period: CD-R highest sensitivity.

**What are the implications of the main findings?**

- Provide guidelines for suitable grating structures and metal choice in GC-SPR.
- Recommend front-side illumination for robust and reproducible GC-SPR sensing.
- Highlight back-side limits: substrate loss/interference reduces resonance clarity.
- Demonstrate RCWA as a predictive tool for GC-SPR sensor design.

**Abstract:**

Commercial optical discs are used as low-cost grating substrates for fabricating grating-coupled surface plasmon resonance (GC-SPR) sensors, and the effects of front-side and back-side illumination are systematically compared. Three different discs were used as grating substrates with grating periods (Λ) of 322 ± 5.2 nm for BD-R, 805 ± 7.5 nm for DVD-R, and 1.582 ± 0.013 µm for CD-R. Silver (Ag) and copper (Cu) films were deposited by magnetron sputtering to form plasmonic gratings. The shallow grating height of BD-R supported continuous metal coverage, while the deeper DVD-R and CD-R grooves resulted in a less continuous layer. Plasmonic responses were measured using wavelength-modulated SPR spectroscopy and predicted with rigorous coupled wave analysis (RCWA). Ag-coated gratings produced sharper and more clearly identifiable resonances than Cu-coated gratings, which exhibited broader due to stronger damping. Front-side illumination produced stronger and more reproducible SPR excitation across all disc types, whereas back-side illumination resulted in more complex spectra as light propagates through the polycarbonate layer. Refractive index sensitivities based using Ag-coated discs of 394, 321, and 290 nm/RIU were obtained for CD-R, BD-R, and DVD-R, respectively. The results clarify the influence of fabrication strategy, illumination geometry, and disc grating geometry on resonance quality and sensitivity in low-cost optical disc-based GC-SPR sensors.

## 1. Introduction

Surface plasmon resonance (SPR) is a powerful optical sensing technique capable of label-free, real-time detection of changes in refractive index at a metal–dielectric interface. Due to its high sensitivity and versatility, SPR biosensors have been extensively applied to biomedical diagnostics [1,2,3], drug screening [4,5,6], and environmental monitoring [7,8]. The traditional SPR systems are based on prism-coupled configurations, especially the well-known Kretschmann setup. In this arrangement, p-polarized light is passed through a high-index prism and directed onto a thin metal layer at varying incident angles to achieve momentum matching with the oscillation-free electron [9,10]. Although this configuration provides a good reflectivity signal and highly sensitive change, it has several drawbacks, including the need for precise angular control, complex optical alignment, and bulky components. So, prism-based systems are less suitable for portable or integrated sensing platforms. To overcome the limitations of prism-based and bulky systems, the grating-coupled SPR (GC-SPR) configuration has been investigated as an alternative. This configuration satisfies the resonance condition through the grating diffraction [9,11]. Therefore, GC-SPR can be implemented in compact and potentially portable designs because it offers practical advantages in size, alignment simplicity, and integration flexibility, making it attractive for miniaturized and portable sensing platforms [12,13,14,15].

Grating fabrication is challenging and is typically performed using techniques such as electron-beam lithography [16], interference lithography [17], or focused ion beam (FIB) milling [18]. These approaches require specialized equipment and high-cost operation. Fabricating large-area gratings also increases processing time and can limit throughput. A low-cost alternative is to repurpose commercial optical discs such as Blu-ray Disc Recordable (BD-R), Digital Versatile Disc Recordable (DVD-R), and Compact Disc Recordable (CD-R). These discs are mass-produced and contain uniform submicron-scale grating patterns with good consistency over large areas. Optical discs also provide a sustainable option by reusing existing materials. The three-disc formats provide different grating geometries, including grating period and groove depth. These parameters determine the additional momentum supplied by the grating and therefore affect surface plasmon excitation in GC-SPR performance [19,20]. Some studies have reported that, after cutting or peeling the disc, the original metal layer used for data storage can remain intact and still support plasmonic excitation without additional coating or cleaning [21,22,23]. However, using the native disc metal layer directly can introduce reproducibility challenges. The metal composition, thickness, and surface condition can vary across disc brands, batches, and sample preparation steps, which can affect resonance quality and measurement repeatability.

Regarding illumination side, front-side illumination is commonly used in optical disc-based GC-SPR studies, where the incident light passes through the analyte solution before reaching the metal-coated grating. This geometry is simple to implement and compatible with many sensing layouts. However, because SPR sensing relies on refractive index changes at the metal–dielectric interface, absorption or scattering in the solution can distort the incident field and affect spectral accuracy. Back-side illumination has therefore been explored as an alternative approach, in which light enters through the transparent substrate and excites surface plasmons at the metal–dielectric interface on the opposite side [24,25,26]. While a limited number of studies have examined front-side and back-side illumination on the same substrate, systematic evaluation of back-side excitation in optical disc-derived GC-SPR platforms remains limited.

Rigorous coupled-wave analysis (RCWA) is a semi-analytical solution method for Maxwell’s equations in periodic structures and is well established for modeling diffraction gratings and plasmonic devices [27,28]. By expanding the fields into Fourier harmonics, RCWA can accurately calculate reflectance spectra, field distributions, and coupling efficiencies for multilayer periodic structures [29,30]. Its efficiency and accuracy make it a powerful tool for predicting SPR behavior and guiding sensor design. Applying RCWA to model optical disc gratings enables quantitative understanding of how grating pitch, illumination geometry, and material properties influence the SPR spectrum.

**Figure 1:**
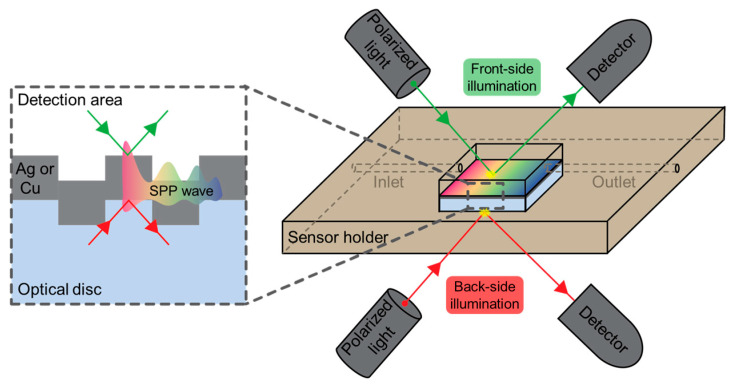
Schematic drawing of the GC-SPR sensor from optical discs, showing the surface plasmon polariton (SPP) wave excitation at the metal–dielectric interface under front-side (green rays) and back–side (red rays) illumination.

In this study, we investigate three types of commercial optical discs (BD-R, DVD-R, and CD-R) as grating substrates for low-cost GC-SPR sensors. Recordable optical discs (-R) types were selected because they provide permanently embossed, uniform grating structures and allow more reliable surface preparation. Each disc format has a distinct grating period and groove depth, providing a set of related structures for examining how grating geometry influences the SPR spectrum and surface plasmon dispersion. Rather than selecting a single disc as a representative grating, the three widely available recordable formats are examined side by side under identical experimental conditions. The role of illumination geometry is also evaluated by measuring both front-side and back-side excitation on the same gratings, allowing direct assessment of substrate effects on resonance visibility, as shown in Figure 1. RCWA simulations are performed using grating parameters and metal thicknesses taken directly from experimental characterization, enabling quantitative comparison between measured and calculated spectra. The experimental measurements, surface plasmon dispersion analysis, and RCWA modeling provide an integrated view of grating-coupled plasmonic behavior on optical discs and clarify how disc type, grating geometry, and illumination direction influence GC-SPR performance.

## 2. Materials and Methods

### 2.1. Chemicals and Materials

Blu-ray Disc Recordable (BD-R, wholesale, 1–6×, 25 GB) was purchased from Nanchang Kunting Electronic Commerce Co., Inc. through AliExpress (Nanchang, China). Digital Versatile Disc Recordable (DVD-R, PlexDisc TY series, 16×, 4.7 GB) was ordered from Vinpower, Inc. (Alhambra, CA, USA) through Amazon. Compact Disc Recordable (CD-R, Memorex Music, 40×, 700 MB) was obtained from Niigata University, Niigata, Japan. An amount of 70% *v*/*v* of nitric acid (HNO_3_, CAS number: 7697–37–2) and 2-propanol (IPA, CAS number: 67–63–0) were purchased from Sigma Aldrich Co. (St. Louis, MO, USA) Norland Optical Adhesive 61 (NOA 61) and Traydex LED small UV/visible light curing ovens were purchase from Norland Products (Jamesburg, NJ, USA). Pure ethylene glycol (EG, CAS Number: 107–21–1) was purchased from PTI Process Chemicals (Ringwood, IL, USA). All solution was used and diluted in deionized (DI) water.

### 2.2. Grating SPR Sensor-Based Optical Disc Fabrication

BD-R, DVD-R, and CD-R discs were cut into 2.5 × 2.5 cm^2^. For BD-R, the grating structure is located on the shiny surface, exposed after removal of the protective plastic film. Cutting a DVD-R separates it into two distinct layers: a white sheet and a transparent plastic sheet, with the grating structure situated on the inner surface of the latter. In the case of CD-R, the disc remains as a single layer, and the grating structure presents on the shiny surface. To clean the cut discs, each sample was immersed in 70% HNO_3_ for 15 min (BD-R) or 5 min (DVD-R and CD-R) to remove the metallic and organic dye coating layers. The samples were then sequentially cleaned in IPA, detergent with tap water, tap water, and DI water (twice), each for 15 min in an ultrasonic bath. The cleaned discs were dried using a handheld blower and stored in a desiccator until use.

The discs were subsequently cut into 1 × 1 cm^2^ pieces and coated with Ag (Silver Target 99.99%, Kurt J. Lesker company, Jefferson Hills, PA, USA) or Cu (Copper Target 99.99%, Plasmaterials, Pleasanton, CA, USA) on the grating side using a sputtering system (Denton Vacuum Desktop Pro, Denton Vacuum, Moorestown, NJ, USA). The coated discs were then mounted onto a 3D-printed SPR microfluidic holder (Bambu Lab P1S 3D Printer, Bambu Lab, Austin, TX, USA) by using NOA 61 adhesive polymer.

### 2.3. Characterizations

Surface morphologies were examined using atomic force microscopy (AFM, Veeco Multi-mode SPM, Veeco Instruments Inc., Plainview, NY, USA with NanoScope Software analysis, v1.5).

**Figure 2:**
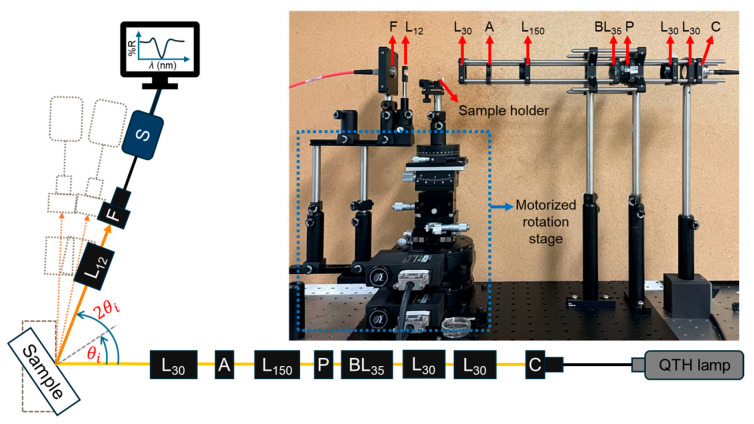
Schematic diagram and photo of the home-built SPR spectrometry-based wavelength-modulation. $\theta_{i}$ denotes the incident angle.

The SPR response was characterized using a home-built SPR spectrometry based on wavelength modulation, as shown in Figure 2. A quartz tungsten–halogen (QTH) lamp with a spectral range of 200–2400 nm (Model 67011, Newport-MKS, Andover, MA, USA) served as the broadband illumination source. The QTH lamp was first connected to the fiber bundle focusing assembly (Model 77776, Newport-MKS) before being linked to a multimode optical fiber (FC–UVIR600–1, Avantes, Apeldoorn, the Netherlands) with a 600 μm core diameter and an FC/PC connector. The fiber output was collimated using a fiber-coupled collimation lens (C) with a focal length (f) of 7.5 mm. This collimator was directly coupled to a plano-convex lens (f = 30 mm, L30) to expand and shape the beam to an approximate spot size of 3 mm at the collimated region. The beam then passed through a series of spherical lenses, including a bi-convex lens (f = 35 mm, BL35), a polarizer, a plano-convex lens (f = 30 mm, L30), a plano-convex lens (f = 150 mm, L30), an aperture, and a final plano-convex lens (f = 30 mm, L30), which focused the beam onto the sample. The lens configuration was selected to minimize the focal spot size at the sample plane while maintaining a practical working distance. The polarizer (P) was used to control the polarization state of the beam and enabled us to select either *p*- or *s*- polarized light, which was important for observing SPR excitation. At the sample position, the focused beam diameter was approximately 0.8 mm. While minor chromatic dispersion resulted in a soft halo around the central focus due to the broadband nature of the light source, this was considered acceptable for the experimental objectives. The surrounding halo could be suppressed using the aperture (A) placed before the final focusing lens. The sample was mounted on a motorized θ – 2θ stage, where the detector arm rotated at twice the angular displacement of the sample stage. The effective incident angle range was limited by the optical beam elongation and obstruction of the optical cage system with the rotation stage. The SPR response was therefore measured over an incident angle range of 30–70°. Reflected light was collected using a plano-convex lens (f = 12 mm, L_12_) and focused onto the detector, which was coupled to a spectrometer (AvaSpec–NXS2048CL–CPI2, Avantes, wavelength range of 300–1100 nm) for SPR signal acquisition and analysis. The data was recorded by varying the $\theta_{i}$ from 30 to 70 by 10 increments under light intensity of *p*-polarization (*I_p_*) and *s*-polarization (*I_s_*). The reflectivity was reported as the relative reflectivity, expressed as the proportion of *I_p_* and *I_s_*.

## 3. Theoretical Description

**Figure 3:**
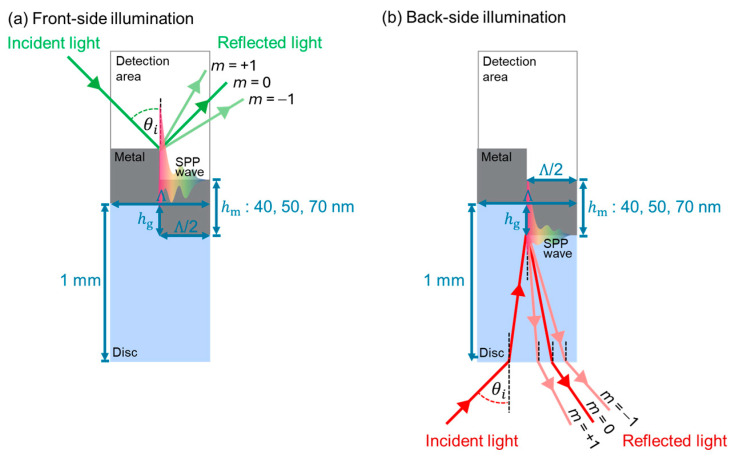
Schematic diagrams of surface plasmon resonance (SPR) excitation using grating-coupled SPR configuration based on metal-coated optical discs: (**a**) front-side illumination and (**b**) back-side illumination. Structure parameters are the grating period (Λ), grating height (*h*_g_), metal film thickness (*h*_m_), and disc thickness (~1 mm). Green and red arrows indicate incident light pathway with incident angle ($\theta_{i}$) and reflected light paths. The rainbow field represents the surface plasmon propagation (SPP) wave.

Two excitation configurations (Figure 3) were examined: front-side and back-side illuminations. In the front-side configuration (Figure 3a), the incident light propagates directly through air, which is taken as the dielectric medium, before reaching the metal-grating interface. Conversely, the back-side illumination (Figure 3b) sets the transparent optical disc substrate toward the light path. This study assumes that each transparent optical disc is composed of polycarbonate. The incident beam first penetrates the disc material before reaching the metal–dielectric interface, where surface plasmon excitation can occur and is responsive to variations in the local refractive index. This comparison enables us to evaluate the effect of the optical pathway on plasmonic coupling and sensor efficacy.

The fundamental mechanism of SPR condition is based on the momentum alignment which established at the metal–dielectric interface between the incident light and the oscillated free electrons of the metal [9,31,32]. The matching momentum determines the excitation of the surface plasmon resonance effect in a particular arrangement. Typically, the momentum of incident light is expressed in terms of the in-plane component of the incident wavevector ($k_{x,inc}$) which is given by:

(1) $$k_{x,inc}=\frac{\omega }{c}n_{d}\sin\theta_{i}$$

where *ω* is an angular frequency, *c* is the speed of light in vacuum (m/s), $n_{d}$ is the refractive index of the incident medium with $n_{d}$ = 1.00 for front-side illumination (air) and $n_{d}$ = 1.54 for back-side illumination (polycarbonate disc substrate), and $\theta_{i}$ is the incident angle [33]. This expression describes the projection of the incident light momentum along the metal surface.

A periodic grating is used to distribute additional momentum components that enable the coupling of free-space photons of the incident light into surface plasmon polaritons. This increment of magnitude momentum (*G*) can be quantitatively characterized as the contribution of the grating to the in-plane wavevector [19,32,34]. By incorporating this effect, the in-plane momentum of incident light parallel to the grating surface can be represented as:

(2) $$k_{\|}=\;k_{x,\mathrm{inc}}+\;mG=\;\frac{\omega }{c}n_{d}\sin\theta_{i}\;\pm m\frac{2\pi }{\Lambda }$$

where Λ denotes the grating period in meters and *m* is the diffraction order.

On the other hand, the surface plasmon polariton has its own characteristic wavevector ($k_{spp}$) as it propagates along the metal–dielectric interface, and it is described by:

(3) $$k_{spp}=\;\frac{\omega }{c}\sqrt{\frac{\varepsilon_{m}\varepsilon_{d}}{\varepsilon_{m}+\;\varepsilon_{d}}}$$

where $\varepsilon_{m}$ and $\varepsilon_{d}$ are the dielectric constants of metal and dielectric medium, respectively.

Surface plasmon resonance condition occurs when the momentum-matching condition is satisfied as $k_{\|}$ = $k_{spp}$. Therefore, the equation can be expressed as: [19,32,34,35]

(4) $$\frac{\omega }{c}n_{d}\sin\theta_{i}\;\pm m\frac{2\pi }{\Lambda }=\frac{\omega }{c}\sqrt{\frac{\varepsilon_{m}\varepsilon_{d}}{\varepsilon_{m}+\varepsilon_{d}}}$$

Within this matching situation, the light couples into surface plasmon phenomena, which along with the reflectance spectrum presents a noticeable drop. The efficiency of the system in exciting plasmons is dependent on the geometry of the grating and the surrounding medium. The optical signal can be linked to the sensor’s structure by monitoring the location and behavior of this resonance. The comparison of front- and back-side illumination enables us to observe the impact of the light path and dielectric surrounding conditions on plasmon excitation.

## 4. Results and Discussion

### 4.1. Structural and Morphological Characterization

**Figure 4:**
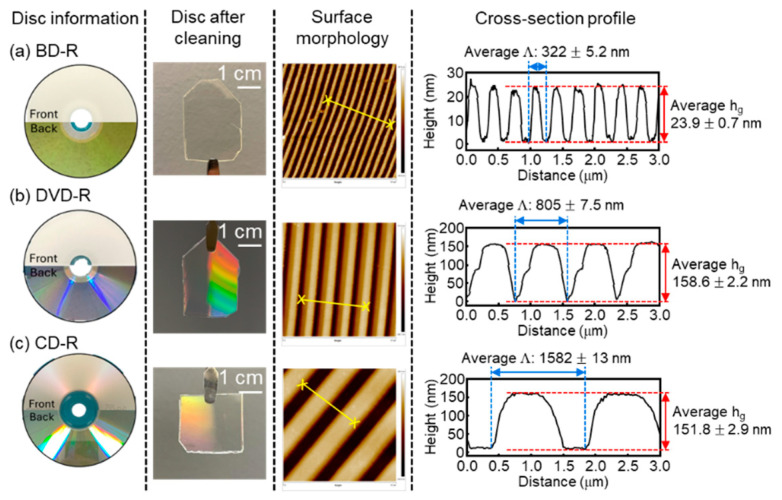
Photos and morphological characterizations of commercial optical discs: (**a**) BD-R, (**b**) DVD-R, and (**c**) CD-R. Each row includes the original disc (front and back), the cleaned optical disc after removal of the metallic and organic dried layers, the AFM topography image (5 µm × 5 µm), and corresponding cross-sectional profiles. In the AFM images, the yellow lines mark the lateral regions used to extract the grating profile. The schematic cross-sections are constructed based on these AFM profiles and define the grating period (Λ) and grating height (*h*_g_).

Each disc type is presented in Figure 4, where panels show (a) the BD-R disc, (b) the DVD-R disc, and (c) the CD-R disc. In each panel, subcolumns provide images of the original disc, the disc after cleaning, the AFM topography, and the corresponding cross-sectional analysis. The reflective metallic coating on the original discs was completely removed using a chemical solution. After cleaning, all substrates appeared as transparent polymer sheets. For the DVD-R and CD-R substrates, diffraction colors were visible to the naked eye, whereas the BD-R substrate did not exhibit an obvious diffraction pattern. To quantify structural differences among the optical discs, AFM images revealed periodic track structures with uniform and well-defined grooves for all disc types. To define the different structure of each optical disc, AFM measurements were averaged over three different locations on each disc (*n* = 3) and are reported as mean ± standard deviation. The BD-R possessed the smallest grating period (Λ), averaging 322 ± 5.2 nm, followed by the DVD-R with an average Λ of 805 ± 7.5 nm, and the CD-R exhibited the largest average Λ of 1.582 ± 13 nm. Cross-section profile analysis further indicated that the BD-R grating height (*h*_g_) was the shallowest, with an average depth of 23.9 ± 0.67 nm. In contrast, the DVD-R and CD-R exhibited comparable average *h*_g_ values of about 158.6 ± 2.2 nm and 151.7 ± 2.9 nm, respectively. In addition to differences in grating period and groove depth, the AFM profiles show that the BD-R grating has a shallower and more rounded groove shape, whereas the DVD-R and CD-R gratings are closer to a rectangular profile. Previous studies have shown that groove profile influences the characteristics of grating-coupled SPR spectra, including resonance depth and linewidth [36]. Therefore, differences in disc groove profile are likely to influence the shape and width of the SPR resonance features. To further examine the influence of metal deposition on surface morphology, AFM measurements were performed on Ag-coated BD-R, DVD-R, and CD-R substrates at different metal thicknesses (scan size 5 µm × 5 µm, see Supporting Information, Figure S1). For clarity, quantitative roughness values are reported here for the CD-R substrate as a representative example. The 10 nm Ag film exhibits a granular morphology, resulting in higher roughness values (*σ*_q_ = 7.32 nm, *σ*_a_ = 5.49 nm). In contrast, the 70 nm Ag film shows a smoother and more continuous surface with substantially reduced roughness (*σ*_q_ = 2.37 nm, *σ*_a_ = 1.88 nm). These observations show that increasing the metal thickness improves film continuity and reduces nanoscale roughness at the interface of metal depositions on polymers. These morphological differences are expected to affect the grating-coupling condition for surface plasmon excitation in the GC-SPR configuration. Furthermore, the experimentally measured structural parameters were used as direct input parameters for the RCWA simulations to enable a consistent experiment-to-simulation comparison.

### 4.2. Study the Plasmonic Effect on Different Optical Disc-Based GC-SPR Sensors

To investigate the plasmonic response of the different grating substrates, each optical disc was coated with two different plasmonic metals (silver (Ag) and copper (Cu)). Ag and Cu were selected to explore alternative plasmonic materials to gold (Au) that are compatible with simpler and lower-cost fabrication routes. Ag is reported as providing sharper plasmonic resonances and higher figures of merit than Au due to its lower intrinsic damping and stronger electromagnetic field confinement [37,38,39,40]. Cu was included as a cost-effective and biocompatible alternative with plasmonic activity extending from the visible to the near-infrared spectral range, which makes it attractive for sensing applications [41,42,43,44].

In addition to metal types, three different metal thickness (*h*_m_ = 40, 50, and 70 nm) were examined to evaluate the influence of *h*_m_ on plasmonic coupling efficiency and film robustness [45,46]. The *h*_m_ in the range of 40 to 50 nm are commonly reported to support efficient surface plasmon excitation. Increasing the thickness to 70 nm approaches optical opacity but the evanescent field can still penetrate the metal film. It can improve film robustness during handling and repeated measurement.

To compare between experimental measurements and RCWA simulations, structural parameters extracted from AFM characterization were used as inputs for the theoretical model. The Λ was set to 320 nm, 800 nm, and 1600 nm for BD-R, DVD-R, and CD-R structures, respectively. The corresponding grating heights (*h*_g_) were set to 25 nm for BD-R, 160 nm for DVD-R, and 150 nm for CD-R. The frequency-dependent dielectric functions of Ag and Cu were taken from Refs. [47,48], while the refractive index of the polymer substrate was assumed to be 1.54 for all disc types [33].

In RCWA, electromagnetic scattering from periodic structures is solved in Fourier space by expanding both the dielectric function and the electromagnetic fields into spatial harmonics [49]. A minimum of 181 Fourier components was used to represent the grating region, which was sufficient to ensure numerical convergence. For front-side illumination, a semi-infinite air region was assumed on the incident side and a semi-infinite optical disc on the transmission side. For back-side illumination, semi-infinite air regions were assumed on both sides, with an additional 1 mm-thick optical disc layer included on the incident side to account for realistic substrate thickness. The inclusion of this layer introduced multimode interference effects, and the simulated spectra were therefore smoothed to more clearly identify the surface plasmon resonance wavelength. Additional details are provided in the Supplementary Information (Figures S2–S5).

#### 4.2.1. Front-Side Illumination

**Figure 5:**
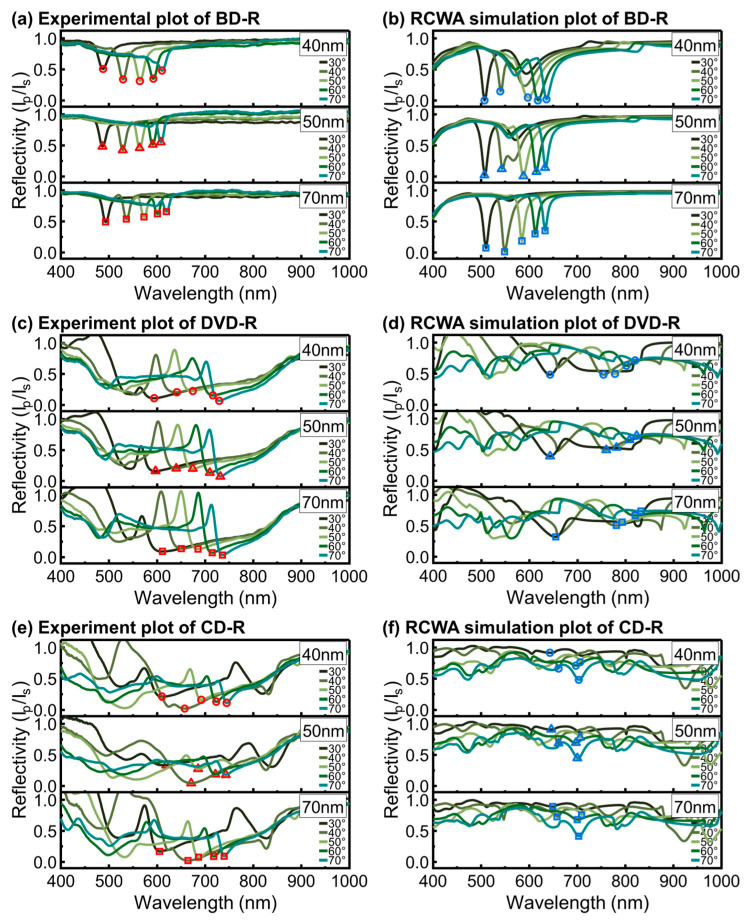
Experimental and RCWA-simulated wavelength-modulated SPR spectra of Ag-coated (**a**,**b**) BD-R, (**c**,**d**) DVD-R, and (**e**,**f**) CD-R under front-side illumination. The extracted resonance wavelengths ($\lambda_{res}$) at each incident angle ($\theta_{i}$) are indicated by symbols, with circles, triangles, and squares corresponding to *h*_m_ of 40, 50, and 70 nm, respectively. The $\lambda_{res}$ of the experiment and RCWA results are shown in red symbols and blue symbols, respectively.

**Figure 6:**
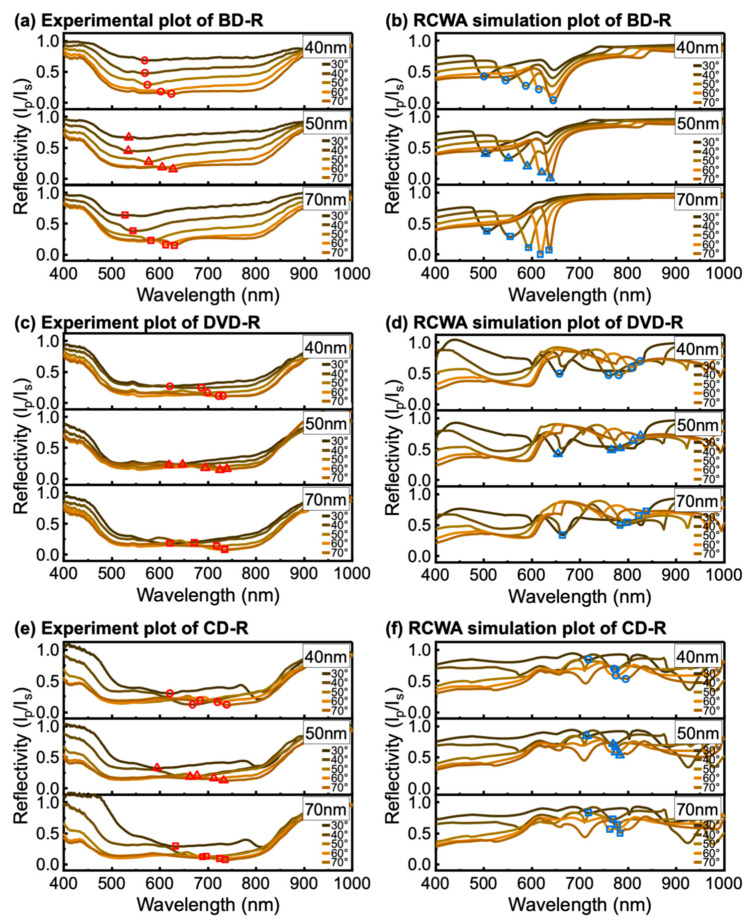
Experimental and RCWA-simulated wavelength-modulated SPR spectra of Cu-coated (**a**,**b**) BD-R, (**c**,**d**) DVD-R, and (**e**,**f**) CD-R under front-side illumination. The extracted resonance wavelengths ($\lambda_{res}$) at each incident angle ($\theta_{i}$) are indicated by symbols, with circles, triangles, and squares corresponding to *h*_m_ of 40, 50, and 70 nm, respectively. The $\lambda_{res}$ of the experiment and RCWA results are shown in red symbols and blue symbols, respectively.

The plasmonic response under front-side illumination was investigated by systematically measuring the SPR spectra of Ag-coated gratings in Figure 5 and Cu-coated gratings in Figure 6 for BD-R, DVD-R, and CD-R.

For Ag-coated discs, the experimental spectra shown in Figure 5a,c,e are compared with the corresponding RCWA simulations in Figure 5b,d,f. Across all disc types, the resonance wavelength ($\lambda_{res}$) at each $\theta_{i}$ exhibited close agreement between experiment and simulation when AFM-derived parameters were used. This agreement supports the reliability of the modeling approach from the RCWA prediction.

For Ag-coated BD-R, both experimental (Figure 5a) and simulated (Figure 5b) spectra exhibited sharp and well-defined resonance dips. These resonances peaks were more distinct than those observed for DVD-R and CD-R. This behavior is primarily attributed to the smaller grating period (Λ) of the BD-R structure, which determines the momentum-matching condition for surface plasmon polariton excitation. Within this fixed-period framework, the relatively shallow *h*_g_ of the BD-R further improves resonance definition by maintaining coupling conditions rather than acting as the dominant parameter controlling the resonance wavelength, consistent with established grating-coupled SPR models [28,50]. The shallow *h*_g_ also allows the deposited Ag film to remain laterally continuous across the grating profile when the *h*_m_ exceeds *h*_g_, enabling partial metal bridging between adjacent ridges [51]. As a result, variations in Ag thickness from 40 to 70 nm produced minor changes in resonance ratio intensity and peak quality, while the resonance wavelength shifted systematically with increasing $\theta_{i}$, confirming that the resonance position is predominantly governed by grating period and illumination geometry rather than groove depth alone.

Ag-coated DVD-R (Figure 5c,d) and CD-R (Figure 5e,f) also exhibited red shifts in $\lambda_{res}$ with increasing $\theta_{i}$, following the same trend as that observed for BD-R. However, the resonance features were broader and less distinct in both experimental and simulated spectra. The experimental resonance dips were observed across different regions of the wavelength range for DVD-R and CD-R. RCWA was used to simulate general shift trends and to estimate resonance characteristics in comparable regions, although the predicted positions did not fully coincide with the experimental values. Compared with BD-R, DVD-R and CD-R have larger *h*_g_. The deeper grooves can reduce film continuity across the grating profile and weaken field confinement at the metal surface. Between DVD-R and CD-R, the DVD-R spectra retained better-defined minima than the CD-R spectra. The larger Λ of the CD-R reduces the effectiveness of momentum matching because the surface appears flatter to the incident wave. This reduces the selectivity of phase matching and broadens the resonance features. In these present measurements, the CD-R configuration produced the lowest spectral sharpness under front-side illumination.

In contrast to Ag-coated samples, Cu-coated discs exhibited significantly broader resonance features in the experimental spectra for all disc types, as shown in Figure 6a,c,e. The corresponding RCWA simulations in Figure 6b,d,f produced sharper and more distinct resonances. This discrepancy arises because RCWA assumes idealized structures with smooth metal films, uniform material properties, and no surface oxidation. In practice, Cu rapidly oxidizes in air and forms surface layers of Cu_2_O and CuO that increase surface roughness and introduce additional optical loss due to inhomogeneity [52]. In addition, the presence of an oxide layer leads to broader SPR resonances and a shift toward higher angles, resulting in a reduced figure of merit [53]. These effects lead to spectral broadening as evidenced in experimental results compared with simulations. Furthermore, the broader resonances observed for Cu-coated gratings can be attributed to the larger imaginary component of the dielectric function of Cu in the visible spectral range. The larger imaginary permittivity causes stronger damping of collective electron oscillations [38,39]. Electromagnetic energy associated with the surface plasmon decays more rapidly within the metal. This behavior shortens the plasmon lifetime and produces shallower and wider SPR dips compared with Ag-coated samples [39]. Thus, the higher intrinsic loss of Cu can contribute to lower resonance contrast as observed in the experimental spectra.

For Cu-coated BD-R gratings (Figure 6a), the SPR dip was weak and often appeared as a near-flat spectral response when $\theta_{i}$ increased. However, the dip remained shallow and was not distinct. In addition, increasing the Cu thickness also improved the visibility of the resonance. The 70 nm of films thickness exhibited more pronounced dips than 40 and 50 nm films. The weak response observed for thinner Cu films indicates inefficient plasmonic coupling. For Cu, the resonance is already strongly damped due to intrinsic absorption, and when the metal layer is too thin, part of the optical energy can leak through the film instead of being confined at the metal–dielectric interface [54]. This combination reduces the resonance contrast, leading to a shallow or nearly flat spectral dip. Increasing the Cu thickness suppresses leakage and enhances field confinement at the interface, so the coupling approaches a better balance between radiative leakage and absorptive loss in the metal film [45]. As a result, the SPR dip becomes more visible and better defined. In this work, the results suggest that Cu-coated discs required a thicker metal layer than Ag-coated gratings to achieve comparable SPR visibility.

On the larger-period substrates (DVD-R in Figure 6c and CD-R in Figure 6d), Cu coatings produced more visible resonance features but still broader than on BD-R. However, the spectral linewidths remained large. Larger grating periods allow momentum matching through higher diffraction orders and provide more diffraction channels for coupling into SPP modes [35]. This can make the resonance dip easier to observe on DVD-R and CD-R even when optical loss of Cu is strong. At the same time, multiple nearby modes can overlap, which reduces spectral selectivity and broadens the resonance.

The front-side illumination results show that grating-coupled SPR excitation can be achieved on optical disc-based substrates using both Ag and Cu coatings. Ag produced sharper and more clearly defined resonance features, while Cu exhibited broader resonances with reduced spectral definition but still supported discernible SPR excitation under appropriate thickness and grating conditions. The difference in resonance quality between Ag- and Cu-coated gratings mainly arises from their intrinsic optical losses. Within the Drude model framework (Figure S6), the imaginary part of the permittivity ($\varepsilon_{m}^{\prime \prime }$) reflects electron damping and energy dissipation. Cu exhibits a larger ($\varepsilon_{m}^{\prime \prime }$) than Ag over the studied wavelength range, resulting in stronger damping and broader SPR resonances. In addition, both Ag and Cu are susceptible to surface degradation and oxidation, which can further reduce resonance quality. Protective layers such as MoS_2_ or graphene have been reported as effective strategies to improve the chemical stability of plasmonic metal films [55,56]. These observations indicate that commercial optical discs provide a practical grating platform for SPR measurements and enable reproducible identification of resonance features across disc types. In addition, this work highlights the feasibility of Ag and Cu as alternative plasmonic materials for low-cost GC-SPR implementations.

#### 4.2.2. Back-Side Illumination

In this study, we considered the back-side illumination geometry, where the incident light enters through the polycarbonate substrate of the optical disc and directly couples to the metal-grating interface from beneath the disc. This configuration was examined as a potential approach to mitigate the effects of scattering or absorption caused by strong solvents in sensing applications [25,26]. Back-side illumination has received limited systematic evaluation in the context of optical disc derived GC-SPR platforms, particularly using matched experimental measurements and RCWA simulations under identical conditions. This configuration was simple to implement. The sample was flipped relative to the front-side geometry, and the same procedure is used to control and align the $\theta_{i}$. The back-side configuration results are presented to clarify how substrate-side coupling influences the SPR response and to provide a complementary perspective to the front-side illumination configuration.

**Figure 7:**
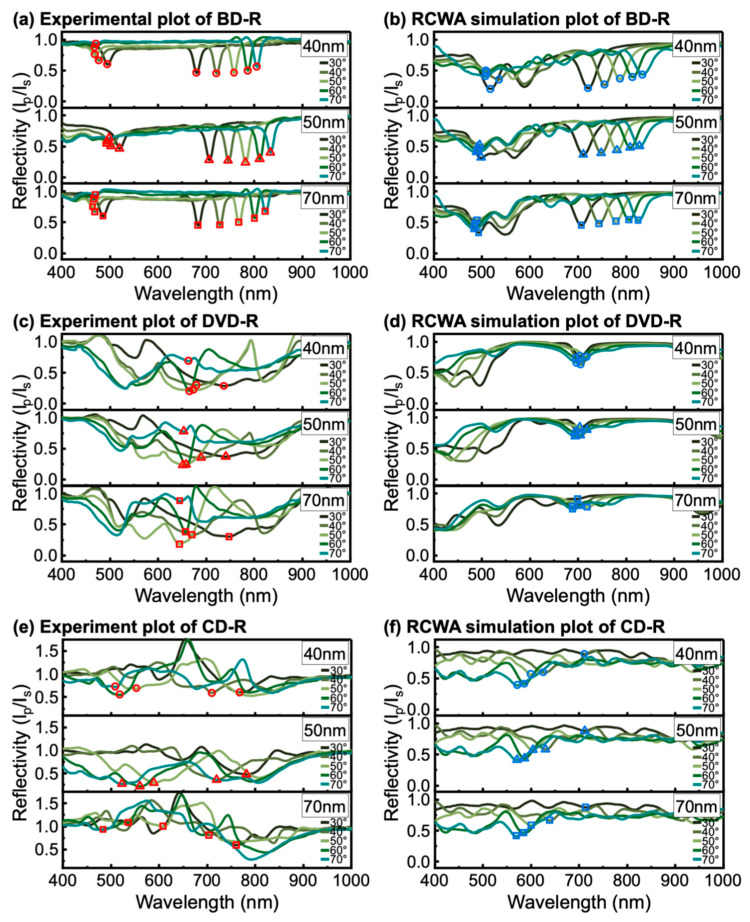
Experimental and RCWA-simulated wavelength-modulated SPR spectra of Ag-coated (**a**,**b**) BD-R, (**c**,**d**) DVD-R, and (**e**,**f**) CD-R under back-side illumination. The extracted resonance wavelengths ($\lambda_{res}$) at each incident angle ($\theta_{i}$) are indicated by symbols, with circles, triangles, and squares corresponding to *h*_m_ of 40, 50, and 70 nm, respectively. The $\lambda_{res}$ of the experiment and RCWA results are shown in red symbols and blue symbols, respectively.

The SPR responses of Ag-coated discs under back-side illumination are shown in Figure 7. For Ag-coated BD-R discs (Figure 7a,b), two groups of SPR dips were observed as the incident angle $\theta_{i}$ increased from 30° to 70°. One group appeared in the shorter-wavelength region (approximately 520 to 471 nm) and blue-shifted with increasing $\theta_{i}$, while the second group appeared in the longer-wavelength region (approximately 680 to 833 nm) and red-shifted with increasing $\theta_{i}$. The excitation wavelength ranges were similar for all Ag thicknesses, indicating that thickness played a minor role in shifting resonance positions under back-side illumination similar to front-side illumination.

The Ag-coated DVD-R (Figure 7c,d) and CD-R spectra (Figure 7e,f) were more difficult to interpret because the resonance features were complex with multiple irregular dips and no clear dominant minimum. In these cases, RCWA simulations were useful for supporting peak assignment. The simulations reproduced the BD-R response well. Two plasmonic dip regions were predicted near 518–484 nm and 711–834 nm. The simulations also indicated where resonances should appear for DVD-R and CD-R. In these cases, the experimental spectra containing multiple overlapping dips could be assigned with high confidence. RCWA therefore provided useful guidance for identifying resonance regions, even though the back-side spectra for DVD-R and CD-R were more complex than those of BD-R.

The clearer SPR response of Ag-coated BD-R under back-side illumination is likely related to its grating geometry. BD-R has a smaller *h*_g_. The metal coating can partially bridge adjacent ridges and create a metal overlap layer. This geometry can facilitate more effective coupling of the electromagnetic field at the metal surface [51]. BD-R also has the smallest Λ. Thus, the higher spatial periodicity and the uniform track structure support more well-defined coupling behavior. In contrast, DVD-R and CD-R do not form the same metal overlap layer because of their larger *h*_g_. Their larger Λ can also promote multiple diffraction contributions, which complicates the resonance response. Furthermore, in the back-side configuration, the incident light enters the sample through the polycarbonate substrate. The incident angle is defined in air, but the propagation angle inside the substrate changes due to refraction. As a result, the effective in-plane wavevector at the metal interface differs from that defined by the external angle. This mismatch can perturb the phase-matching condition and disturb the angular region associated with total internal reflection. These effects reduce resonance contrast and make the dip positions more difficult to identify for DVD-R and CD-R.

**Figure 8:**
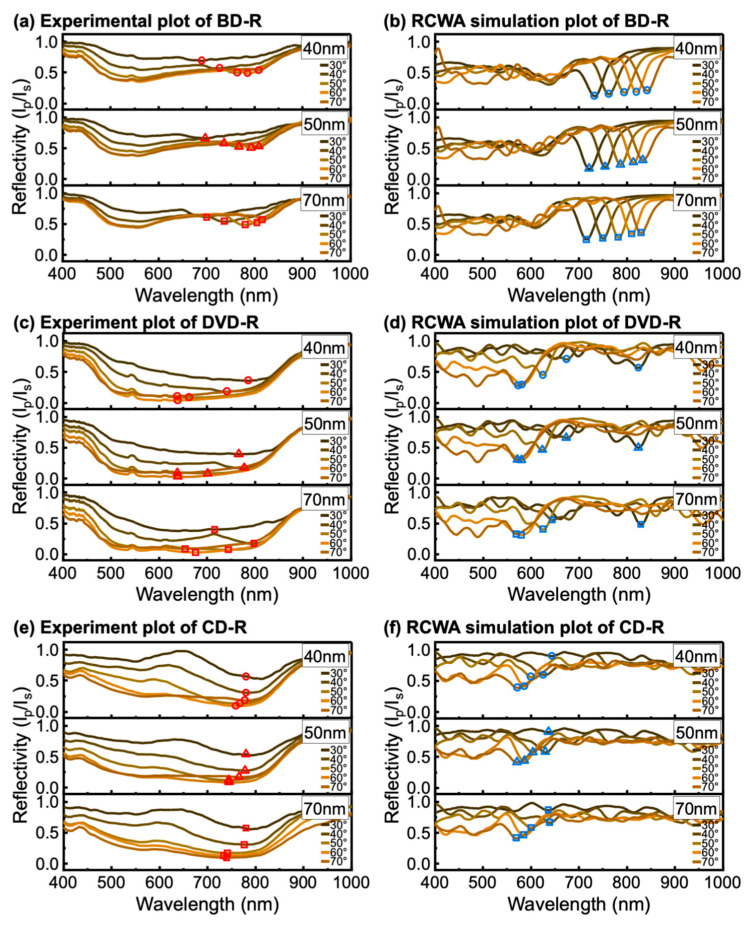
Experimental and RCWA-simulated wavelength-modulated SPR spectra of Cu-coated (**a**,**b**) BD-R, (**c**,**d**) DVD-R, and (**e**,**f**) CD-R under back-side illumination. The extracted resonance wavelengths ($\lambda_{res}$) at each incident angle ($\theta_{i}$) are indicated by symbols, with circles, triangles, and squares corresponding to *h*_m_ of 40, 50, and 70 nm, respectively. The $\lambda_{res}$ of the experiment and RCWA results are shown in red symbols and blue symbols, respectively.

Cu-coated discs under back-side illumination are shown in Figure 8. The measured spectra were broadened similar to the front-side configuration. In the BD-R case (Figure 8a,b), the resonance shifts with incident angle followed the RCWA predictions reasonably well. The DVD-R and CD-R cases (Figure 8c,f) were harder to interpret because the dips were weak and difficult to separate from background variation, and RCWA simulations (Figure 8d for DVD-R and Figure 8f for CD-R) were therefore used to guide resonance assignment. In these larger-period cases, the experimental spectra contained no clear dominant dip that could be identified. The resonance assignment was therefore less particular than for BD-R. Cu-coated gratings already exhibited broad and low-contrast features under front-side illumination, which limited accurate determination of $\lambda_{res}$. Under back-side illumination, propagation through the polycarbonate substrate alters the illumination and collection geometry and perturbs the effective incidence condition at the metal–dielectric interface. To evaluate whether this behavior is related to material absorption, the transmission spectra of the bare disc substrates were measured. As shown in Figure S7, all discs exhibit high optical transmission across the visible spectral range, indicating negligible absorption by the polycarbonate substrate at the wavelengths relevant to this work. Therefore, the reduced resonance contrast observed under back-side illumination is attributed to the gratings’ geometric effects associated with propagation through a millimeter-thick substrate, rather than absorption within the polycarbonate substrate itself. The plasmonic excitation is therefore further suppressed in the experimental spectra. The dips were nearly absent in several measurements, particularly for DVD-R and CD-R.

This study found that back-side illumination reduced resonance visibility. Interference within the substrate weakened the electromagnetic field reaching the metal–dielectric interface, and the effective phase-matching condition deviated from the external incident angle. As a result, only BD-R showed reasonable agreement between experiment and RCWA under back-side illumination, while DVD-R and CD-R often exhibited complex spectra with no clear dominant minima. These results indicate that front-side illumination is the preferred geometry for practical optical disc-based GC-SPR sensing. Back-side illumination may still be useful for measurements involving solvent exposure, but it requires additional optical and mechanical considerations. Substrate thickness should be addressed explicitly. Reducing the polycarbonate thickness may increase the field strength at the metal–dielectric interface and improve plasmon excitation. Another approach is to incorporate a prism on the substrate side to enhance coupling and provide additional excitation pathways. Prism–grating hybrid configurations have been reported [57,58,59]. The presence of a grating can enhance coupling efficiency and improve sensitivity while reducing solution-side effects.

#### 4.2.3. Surface Plasmon Dispersion Analysis

To clarify the momentum-matching conditions for surface plasmon excitation and to verify the physical origin of the experimentally observed SPR modes, a surface plasmon (SP) dispersion analysis was performed and compared directly with RCWA simulations. In this analysis, the in-plane wavevector was evaluated as a function of incident angle and wavelength, with the additional momentum contribution provided by the grating periodicity, as described by the grating-coupling relations in Section 3 and Equations (1)–(4). Surface plasmon resonance occurs when the grating-assisted in-plane wavevector satisfies the surface plasmon polariton (SPP) dispersion relation at the metal–dielectric interface.

**Figure 9:**
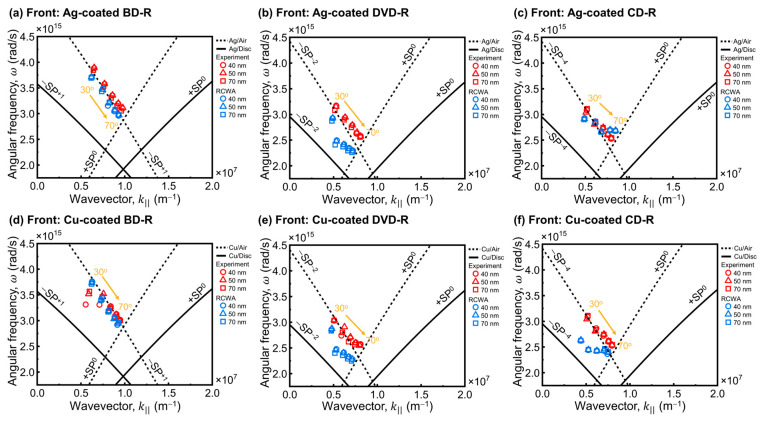
SP dispersion plots for Ag- and Cu-coated optical disc under front-side illumination: (**a**,**d**) BD-R, (**b**,**e**) DVD-R, and (**c**,**f**) CD-R. Red symbols and blue symbols denote $\lambda_{res}$ of experiment and RCWA, respectively. Solid curves represent SP dispersion line occur at the metal-air interface, and dashed curves represent SP dispersion line occur at the metal–disc interface. Yellow arrows indicate the trend of increasing incident angle from 30° to 70°.

**Figure 10:**
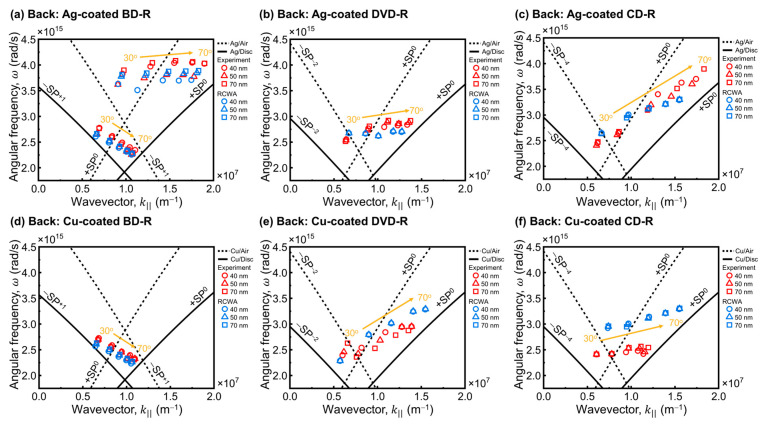
SP dispersion plots for Ag- and Cu-coated optical disc under back-side illumination: (**a**,**d**) BD-R, (**b**,**e**) DVD-R, and (**c**,**f**) CD-R. Red symbols and blue symbols denote $\lambda_{res}$ of experiment and RCWA, respectively. Solid curves represent SP dispersion line occur at the metal-air interface, and dashed curves represent SP dispersion line occur at the metal–disc interface. Yellow arrows indicate the trend of increasing incident angle from 30° to 70°.

The resulting SP dispersion plots are shown in Figure 9 for front-side illumination and Figure 10 for back-side illumination. In each plot, the solid line curves represent the SP dispersion at the metal–air interface, while the dashed line curves correspond to SP dispersion at the metal–disc interface. For clarity, only the SP branches predicted by Equation (4) that correspond to the observed resonance features under the present conditions are shown. Other branches are not displayed because they do not contribute to the measured SPR response. The experimentally extracted $\lambda_{res}$ are plotted as red symbols, and the corresponding RCWA-predicted resonance positions are shown as blue symbols, allowing direct comparison between experiment and simulation.

Comparison between front-side and back-side illumination shows that the illumination geometry affects the strength and clarity of plasmonic excitation rather than the dispersion relation. Under front-side illumination (Figure 9), the resonance points extracted from the experiment align with the SP dispersion branches predicted by RCWA across the disc types. This alignment indicates that grating-assisted momentum matching is strong enough to produce observable resonance features. In this configuration, the resonance dips are generally easier to identify and track as a function of $\theta_{i}$. Resonance dips that are easier to track allow more reliable identification of $\lambda_{res}$ on the corresponding dispersion branches in both the experimental data and the RCWA simulations.

In back-side illumination (Figure 10), the experimental and RCWA resonance points show larger deviations from the SP dispersion curves. BD-R shows the best agreement between experiment and RCWA, while DVD-R and CD-R show weaker and less reproducible resonance features. The broad and less well-defined plasmonic excitation make peak identification difficult and increase uncertainty in determining $\lambda_{res}$. The mismatch between resonance dips and SP dispersion line under back-side illumination can be attributed to light propagation through the millimeter-thick polycarbonate substrate before reaching the metal–grating interface. The substrate changes the effective incidence condition at the interface and reduces the field strength available for plasmon excitation. This study indicates that front-side illumination enables more reliable resonance tracking for dispersion-based mode identification, whereas back-side illumination remains limited by the substrate.

The SP dispersion analysis further reveals a strong dependence of the accessible diffraction order (*m*) on the Λ of the optical disc substrates. For BD-R (Figure 9a,d and Figure 10a,d), which has the smallest Λ, the experimentally observed and simulated resonance points primarily follow the +*SP*^0^ and −*SP*^+1^ branches, indicating that plasmonic coupling occurs predominantly through low diffraction orders within the measured angular range.

As the grating period increases, additional diffraction orders become accessible. For DVD-R (Figure 9b,e and Figure 10b,e), the resonance points follow the +*SP*^0^ and −*SP*^−^^2^ branches, while for CD-R (Figure 9c,f and Figure 10c,d), coupling extends to higher-order branches up to −*SP*^−^^4^ across the visible to near-infrared spectral region. This trend can be explained by the grating momentum (G=2π/Λ), which provides the additional in-plane wavevector needed for coupling. A larger Λ gives a smaller *G*. As a result, a larger |*m*| is required to supply sufficient momentum to match the SPP dispersion within the same wavelength and angular ranges [29]. Therefore, BD-R is associated mainly with low-order branches, whereas DVD-R and CD-R exhibit higher-order branches in the dispersion plots.

Among the three discs, CD-R supports the most significant number of accessible diffraction orders, followed by DVD-R and then BD-R. This trend is consistent with the diffraction colors observed on the cleaned DVD-R and CD-R substrates under ambient illumination, whereas BD-R does not exhibit pronounced diffraction effects. Although larger-period gratings can enable access to additional diffraction orders, the coupling condition can become less selective within the measured wavelength and angular ranges. As a result, multiple nearby grating-assisted solutions may contribute to the measured spectra, which can broaden the resonance features and reduce the clarity of the observed SPR dips.

For a given grating geometry and illumination condition, the overall shape of the SPP dispersion relation remains similar for Ag- and Cu-coated gratings. Although the plasma frequency of Cu is lower than that of Ag, the difference in the dispersion relation becomes less pronounced when expressed in terms of angular frequency. Consequently, the SPP dispersion of Cu can be regarded as a small perturbation of that of Ag, and the diffraction-order assignments remain largely unchanged between the two metals.

Accordingly, the dominant resonance branches correspond to −*SP*^+1^ for BD-R, −*SP*^−^^2^ for DVD-R, and −*SP*^−^^4^ for CD-R for both Ag and Cu coatings. The primary difference between the two metals lies in the resonance linewidth and visibility rather than in the momentum-matching condition. As discussed in Section 4.2.1 and Section 4.2.2, the higher intrinsic optical loss of Cu leads to broader and weaker resonance features compared with Ag. The dispersion analysis confirms that grating geometry and illumination configuration determine the accessible plasmonic modes, whereas optical loss in the metal film controls resonance sharpness and dip visibility.

## 5. Sensitivity Study

**Figure 11:**
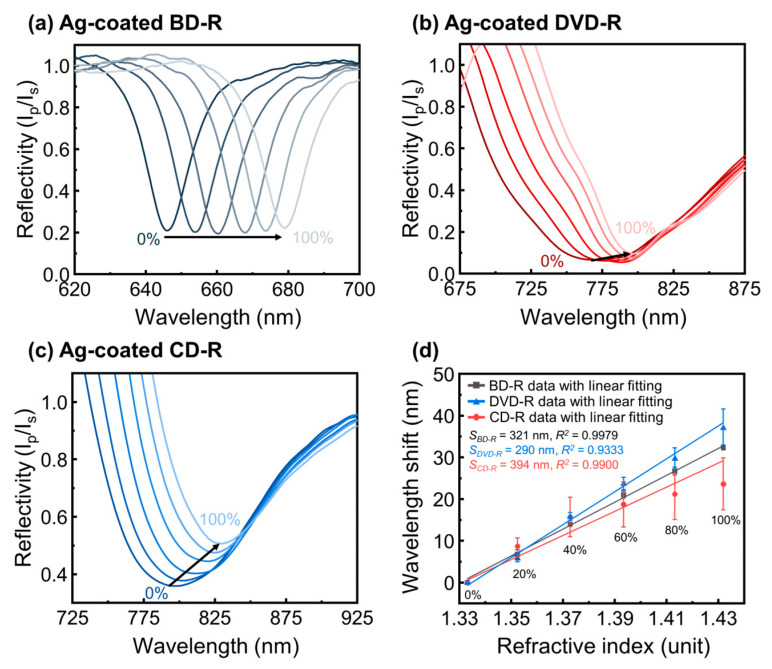
SPR reflectance spectra of using Ag-coated (**a**) BD-R, (**b**) DVD-R, and (**c**) CD-R based GC-SPR sensors measured at different refractive indices using variant concentration of ethylene glycol-water solutions (0–100%*w*/*w*). Black arrows show the increasing concentration trend. (**d**) SPR wavelength shift ($∆\lambda_{res}$) versus refractive index unit (RIU) for BD-R, DVD-R, and CD-R, with linear fitting lines used to determine sensitivity (*S*).

The refractive index (RI) sensitivity of the optical disc-based GC-SPR sensors was evaluated using Ag-coated gratings. Ag was selected for the sensitivity study because it consistently provided sharper and more clearly identifiable resonance features than Cu and more effective extraction of the $\lambda_{res}$ shift. The RI response was examined by varying the surrounding RI using ethylene glycol–aqueous solutions with concentrations ranging from 20 to 100 (%*w*/*w*) [60]. The SPR reflectance spectra was recorded at fixed 50° of $\theta_{i}$ and the $\lambda_{res}$ was determined from the reflectivity minimum. Representative SPR spectra for BD-R, DVD-R, and CD-R substrates are shown in Figure 11a–c, respectively. For all disc types, increasing the RI produced a systematic red shift of the $\lambda_{res}$, confirming that the observed spectral changes originate from RI variations at the metal–dielectric interface. BD-R maintained well-defined resonance dips across the observed wavelength range. DVD-R and CD-R also exhibited clear spectral responses, although some spectral regions showed high reflectivity due to the use of the intensity ratio $I_{p}/I_{s}$, which can amplify background variations in broader resonances.

The extracted resonance wavelength shifts ($∆\lambda_{res}$) were summarized using the resonance wavelength in deionized water (0%*w*/*w*) as the reference. Each disc measurement was repeated three times (*n* = 3). The $∆\lambda_{res}$ was plotted as a function of RI for each disc type, as shown in Figure 11d. Linear regression was applied to determine the RI sensitivity (*S* in nm/RIU), defined as [32]

(5) $$S=\frac{∆\lambda_{res}}{∆n}$$

where *n* is the refractive index of the ethylene glycol.

Among the three substrates, the CD-R–based GC-SPR sensor exhibited the highest sensitivity, with *S* = 394 nm/RIU (*R*^2^ = 0.9900), followed by BD-R with *S* = 321 nm/RIU (*R*^2^ = 0.9333), and DVD-R with *S* = 290 nm/RIU (*R*^2^ = 0.9979). The higher sensitivity observed for CD-R can be understood in terms of its grating geometry and coupling behavior. As discussed in Section 4.2.3, the larger grating period of CD-R supports higher-order and less selective grating-assisted coupling, which results in broader resonance features. Although these broader dips reduce spectral sharpness, the $∆\lambda_{res}$ was wider with changes in RI, leading to a larger measured sensitivity. In contrast, BD-R, which has the smallest Λ, produces sharper and more well-defined resonance features but shows a slightly smaller $∆\lambda_{res}$ compared with CD-R. However, BD-R exhibited the highest measurement consistency, as reflected by the smallest error bars, whereas DVD-R showed the lowest sensitivity and the most variation among repeated measurements.

These results demonstrate a clear relationship between resonance sharpness and refractive index sensitivity in optical disc-based GC-SPR sensors. Smaller grating periods support more distinct resonance features, while larger grating periods produce broader peaks and larger resonance shifts with refractive index. The observed sensitivity trend agrees with the plasmonic response and dispersion analysis discussed above. These results confirm that commercial optical discs provide a potential for fabricating a low-cost SPR platform whose sensing performance can be adjusted through appropriate selection of disc geometry.

## 6. Conclusions

This work provides a systematic evaluation of commercial optical discs as grating platforms for GC-SPR sensing. Comparing BD-R, DVD-R, and CD-R under identical measurement conditions, with RCWA as a quantitative reference, identifies the key roles of grating geometry, illumination configuration, and plasmonic metal selection in determining resonance visibility and sensor performance. The results show that the grating period is the dominant structural parameter controlling coupling behavior and accessible diffraction orders. BD-R, with the smallest period, produced the sharpest and most clearly identifiable SPR excitation and showed the closest agreement between experiment and simulation. DVD-R and CD-R, with larger grating periods, exhibited progressively broader spectral responses. A direct comparison of illumination geometries further indicates that front-side illumination provides stronger and more reliable SPR excitation than back-side illumination. Back-side illumination is limited by light loss and interference in the polycarbonate layer, which reduces resonance visibility for larger-period gratings. Among the plasmonic metals investigated, Ag produced sharper and more clearly defined resonance features than Cu due to its lower intrinsic damping, making Ag a practical low-cost alternative to Au for this optical disc-based GC-SPR platform. Although Cu could support SPR excitation when thicker films were used, its higher optical loss resulted in weaker and broader responses. Therefore, integrating optical disc substrates into GC-SPR platforms demonstrated in this work underscores the importance of considering both substrate properties and illumination geometry when aiming to optimize sensor performance. When making efficient and reproducible GC-SPR sensors, the geometry of the light should be seen as an important design factor.

## Data Availability

The original contributions presented in this study are included in the article/Supplementary Materials. Further inquiries can be directed to the corresponding authors.

## Supplementary Materials

The following supporting information can be downloaded at: https://www.mdpi.com/article/10.3390/ma19030603/s1, Figure S1: AFM topography images of Ag- (Top) and Cu-coated (Bottom) on (a,b) BD-R, (c,d) DVD-R, and (e,f) CD-R gratings deposited with nominal metal thicknesses of 10 nm and 70 nm (scan size: 5 × 5 µm^2^); Figure S2: Comparison between the results for front-side illumination of silver and the BD-R device: (a) experiment, (b) simulation for semi-infinite BD-R, and (c) simulation for 1 mm BD-R; Figure S3: Comparison between the results for back-side illumination for Silver and BD-R device: (a) experiment, (b) simulation for semi-infinite BD-R, (c) simulation for 1 mm BD-R, and (d) simulation with smooth data of 1 mm BD-R; Figure S4: Simulation results varying the diffraction orders from 1 to 100 for front illumination: (a) Ag-coated BD-R structure, (b) Ag-coated DVD-R structure, and (c) Cu-coated CD-R structure; Figure S5: The change in data for different window sizes considered for (a) movemean filter, (b) the Savitzky-Golay filter; Figure S6: The calculated (a) real and (b) imaginary parts of the permittivity for Ag and Cu, respectively, based on the Drude model over the wavelength range relevant to this study [27,28,61,62]; Figure S7: Transmittance spectra of the bare BD-R, DVD-R, and CD-R substrates; Table S1: Drude parameters for Silver and Copper.

## Abbreviations

The following abbreviations are used in this manuscript:

SPR	Surface Plasmon Resonance
SPP	Surface Plasmon Polaritons
GC-SPR	Grating-coupled Surface Plasmon Resonance
BD-R	Blu-ray Disc Recordable
DVD-R	Digital Versatile Disc Recordable
CD-R	Compact Disc Recordable
Au	Gold
Ag	Silver
Cu	Copper
RCWA	Rigorous coupled-wave analysis
FDTD	Finite-Difference Time-Domain
AFM	Atomic Force Microscopy
cm	Centimeter
mm	Millimeter
nm	Nanometer
μm	Micrometer
HNO_3_	Nitric acid
IPA	Isopropanol
DI water	Deionized water
EG	Ethylene glycol
f	Focal length in millimeter
$\theta_{i}$	Incident angle
*I*_p_	Intensity of *p*-polarization
*I*_s_	Intensity of *s*-polarization
Λ	Grating period
*h*_g_	Grating height
*h*_m_	Metal thickness
*m*	Diffraction order
*G*	Grating momentum
*ω*	Angular frequency
*c*	The speed of light in vacuum (m/s)
m/s	Meter per second
$n_{d}$	Refractive index of the incident medium
n or RI	Refractive index of dielectric medium or solution
$\varepsilon_{m}$	Dielectric constants of metal
$\varepsilon_{d}$	Dielectric constants of dielectric medium
$k_{x}$	The in-plane component of the incident wavevector
$k_{spp}$	The wavevector of the SPP propagation along the metal–dielectric interface
$k_{\|}$	The total in-plane wavevector for plasmon excitation
S	Sensitivity (nm/RIU)
*σ*	Surface roughness (nm)
$\lambda_{res}$	Resonance wavelength (nm)
