# Supplementary material for: Double-Sided Illumination Grating-Coupled Surface Plasmon Resonance Sensors Using Direct Optical Discs
Source: Materials (Basel). 2026 Feb 4;19(3):603. doi: 10.3390/ma19030603 (PMC12898446; doi:10.3390/ma19030603)
Supplement: Supplementary file 1 [file materials-19-00603-s001.zip › materials-4090605-supplementary.pdf]

Supplementary Materials

Double-Sided Illumination Grating-Coupled Surface Plasmon Resonance Sensors using Direct Optical Discs

Wisansaya Jaikeandee [1], Asad Ullah Hil Gulib [1], Taeyul Choi [1,*], and Richard Z. Zhang [1,*]

[1]Department of Mechanical Engineering, University of North Texas, Denton, 76207, USA; wisansaya.jaikeandee@unt.edu
******Correspondence:* zihao.zhang@unt.edu (R.Z.Z.); tae-youl.choi@unt.edu (T.C.)

AFM characterization of Ag- and Cu-coated disc gratings:

For all disc types in Figure S1, increasing metal thickness results in visibly smoother surface morphology. The underlying grating structure remains clearly resolved after metal deposition, indicating conformal coating on the polymer substrates.

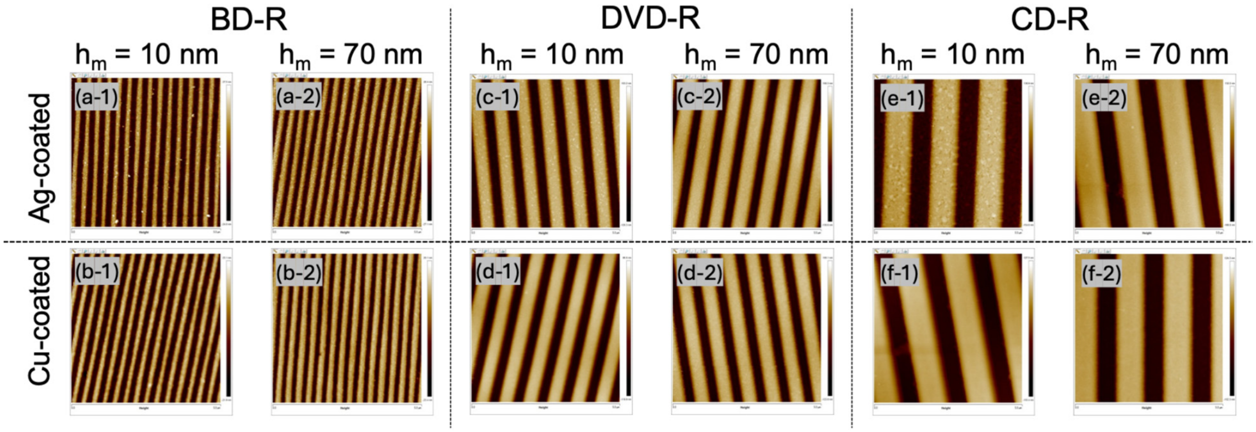

Figure S1. AFM topography images of Ag- (Ttop) and Cu-coated (bBottom) on (a, b) BD-R, (c, d) DVD-R, and (e,f) CD-R gratings deposited with nominal metal thicknesses of 10 nm and 70 nm (scan size: 5 × 5 μm²).

RCWA simulation conditions:

Figure S2. compares the experimental data and simulated results for a silver–BD-R structure under front-side illumination, considering both semi-infinite BD-R and 1 mm-thick BD-R on the transmission side. The results show that all three cases produce similar spectral behavior, with only minor differences arising from interference effects when the 1 mm BD-R layer is included. Therefore, semi-infinite BD-R, DVD-R, and CD-R substrates were considered for front-side illumination in the subsequent simulations.

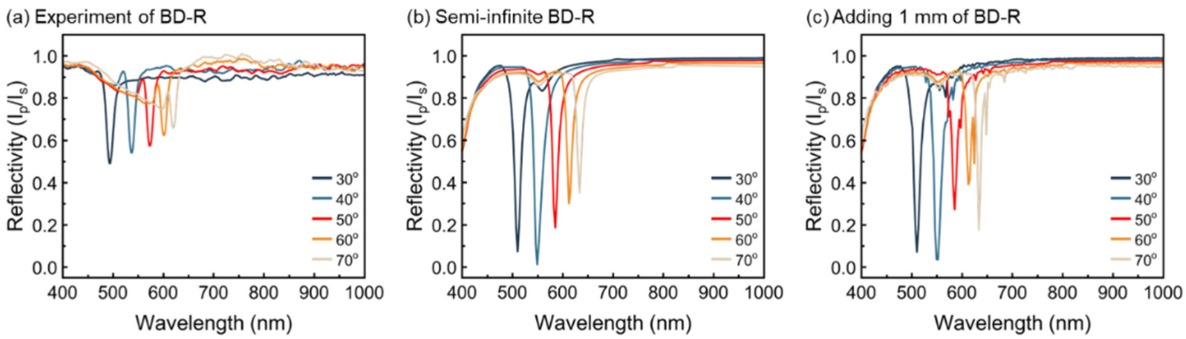

Figure S2. Comparison between the results for front-side illumination of silver and the BD-R device: (a) experiment, (b) simulation for semi-infinite BD-R, and (c) simulation for 1 mm BD-R.

Figure S3~~.~~ presents the comparison between experimental and simulated reflectance spectra for the silver–BD-R structure under back-side illumination. When using a semi-infinite BD-R substrate, the simulated resonant dips are slightly right-shifted relative to the experimental data. However, when a 1 mm-thick BD-R layer is considered on the incident side with semi-infinite air as the incident medium, the simulated dips align closely with the experimental results. In this case, multiple-mode interference within the 1 mm BD-R layer introduces noise in the spectra. To clearly identify the surface plasmon resonance (SPR) dips, a data-smoothing technique was applied. This method is used in data analysis to minimize interference noise and enhance the visibility of key spectral features.

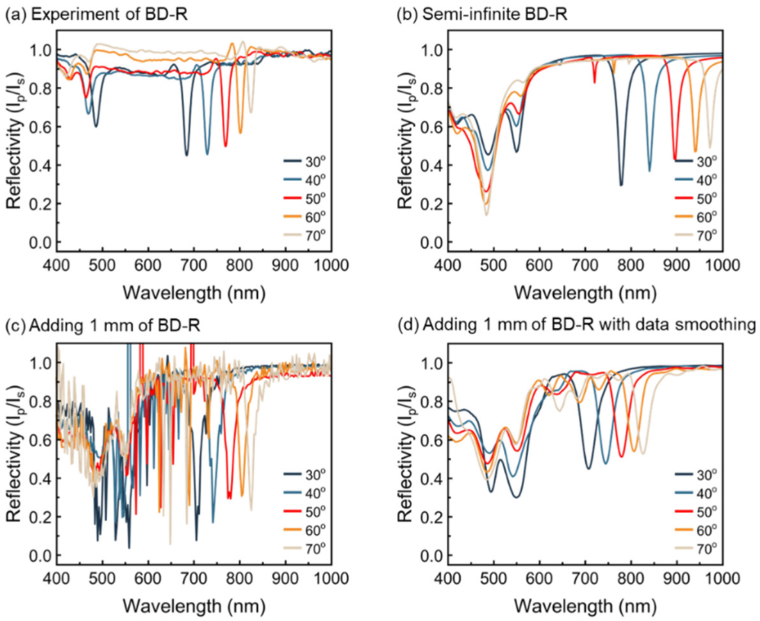

Figure S3. Comparison between the results for back-side illumination for s~~S~~ilver and BD-R device: (a) experiment, (b) simulation for semi-infinite BD-R, (c) simulation for 1 mm BD-R, and (d) simulation with smooth data of 1 mm BD-R.

Figure S4. illustrates the convergence behavior of RCWA simulations for Ag-coated BD-R, Ag-coated DVD-R, and Cu-coated CD-R structures at 507 nm, 655 nm, and 930 nm, respectively, under TM polarization and front illumination. The number of diffraction orders was varied from 1 to 100. The results indicate that the BD-R structure converges after approximately 10 diffraction orders, while both DVD-R and CD-R structures achieve convergence after about 30 diffraction orders.

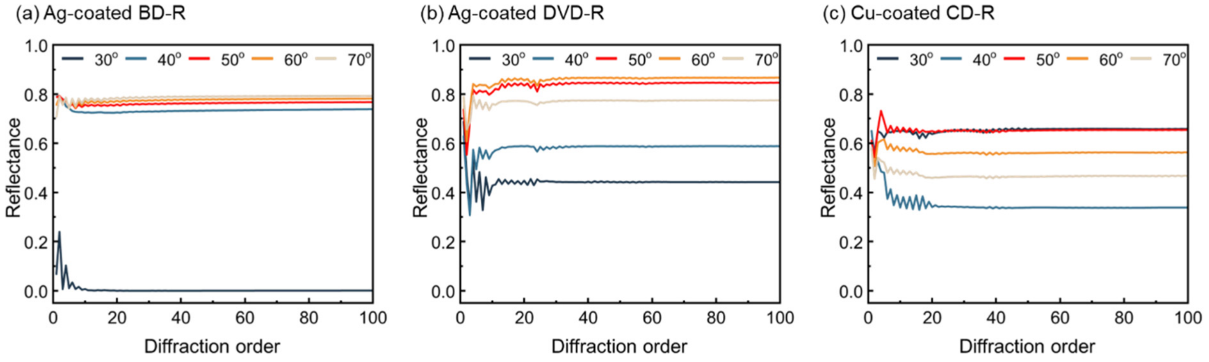

Figure S4. Simulation results varying the diffraction orders from 1 to 100 for front illumination: (a) Ag-coated BD-R structure, (b) Ag-coated DVD-R structure, and (c) Cu-coated CD-R structure.

To reduce numerical artifacts and interference-induced fluctuations in the reflectance spectra, a multi-step smoothing procedure was applied. These artifacts are common in RCWA due to multiple mode interference. First, the raw reflectance data were clamped to a maximum of 1 to enforce physical limits. Then, a spike removal algorithm was employed to suppress sharp outliers by comparing each data point with the average of its immediate neighbors and replacing it if the deviation exceeded 25%. The resulting data were further smoothed using a moving average filter (*movemean* function in MATLAB) with a window size of 9 to suppress high-frequency noise.

To preserve spectral features such as peaks and valleys, a Savitzky–Golay filter (using the *sgolayfilt* function in MATLAB) with a third-order polynomial and an 11-point window was applied. Finally, the smoothed data were interpolated over a finer wavelength grid using spline interpolation to improve visual continuity and enable accurate peak detection. This approach ensures the reflectance curves are both physically consistent and visually comparable to experimental results.

Figure S5. demonstrates the sensitivity of the smoothing process to the number of points used in both the moving-average and Savitzky–Golay filters. The left panel shows results for moving-average windows of 3, 5, 7, 9, 11, and 13 points, where increasing the window size produces smoother curves. The right panel presents results for Savitzky–Golay windows of 5, 7, 9, 11, 13, and 15 points, indicating that the filter is relatively insensitive to window size within this range. All other simulation parameters remained identical to those described previously.

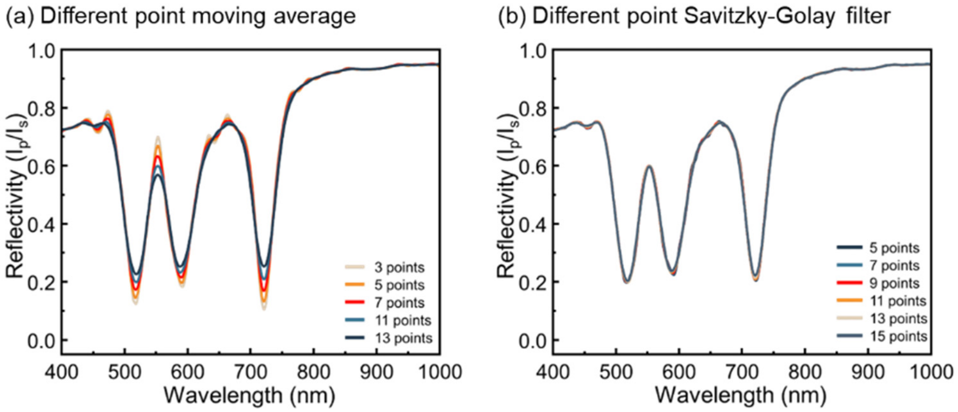

Figure S5. The change in data for different window sizes considered for (a) movemean filter (b) Savitzky-–Golay filter.

Drude model interpretation of the real and imaginary parts of metal permittivity:

The Drude model was employed to describe the free-electron contribution to the complex permittivity. In the Drude formalism, the metal permittivity is expressed as [1,2]

$$\varepsilon_m(\omega) = \varepsilon_\infty - \frac{\omega_p^2}{\omega^2 + i\gamma\omega} \quad (1)$$

where ε_∞ represents the high-frequency contribution, ω_p is the plasma frequency, and γ is the plasma damping rate. The complex permittivity can be written as $\varepsilon_m(\omega) = \varepsilon'_m + i\varepsilon''_m$, where the real part ε'_m describes the dispersive response of the metal and governs its ability to support surface plasmon modes, while the imaginary part (ε''_m) represents dissipative loss associated with electron scattering processes.

By separating the Drude expression into real and imaginary components, the following relations are obtained:

$$\varepsilon'_m(\omega) = \varepsilon_\infty - \frac{\omega_p^2}{\omega^2 + \gamma^2} \quad (2)$$

$$\varepsilon''_m(\omega) = \frac{\omega_p^2\gamma}{\omega(\omega^2 + \gamma^2)} \quad (3)$$

The damping rate is related to the electron relaxation time (τ) through $\gamma = 1/\tau$ [2], such that a larger ε''_m corresponds to a shorter relaxation time and higher optical loss. Consequently, metals with larger ε''_m are expected to exhibit stronger plasmon damping and broader resonance features. The Drude parameters for sSilver and cCopper are given in the following table:

Table S1. Drude parameters for sSilver and cCopper

	ε_∞	ω_p (rad/s)	γ(rad/s)
Silver (Ag) [3]	3.4	1.38×10^{16}	2.7×10^{13}
Copper (Cu) [4]	1	1.64×10^{16}	4.6×10^{13}

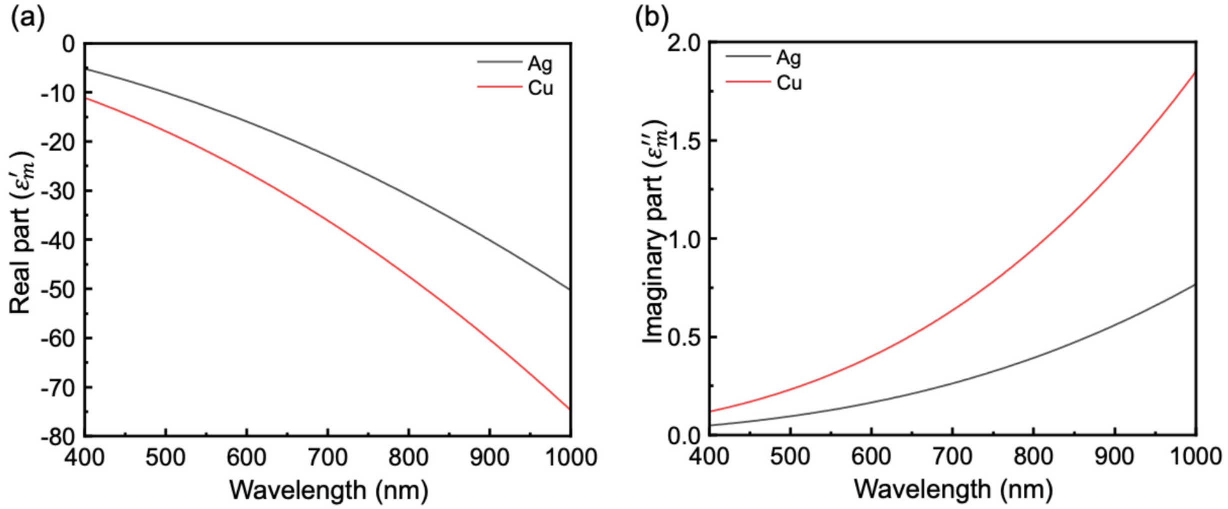

Figure S6 The calculated (a) real and (b) imaginary parts of the permittivity for Ag and Cu, respectively, based on the Drude model over the wavelength range relevant to this study.

References:

[1] Zhang, Z.M. Mechanical Engineering Series (Nano/Microscale Heat Transfer), 2nd ed.; Springer Nature Switzerland, **2020**; pp. 532-636.

[2] Maier, S.A. Plasmonics : Fundamentals and Applications; Springer, **2007**; pp 5-12.

[3] Araki, K.; Zhang, R.Z. Plasmon-Resonance Emission Tailoring of "Origami" Graphene-Covered Photonic Gratings. *Opt. Express* **2020**, *28*, 22791.

[4] Rakic, AD; Djurisic, AB; Elazar, JM; Majewski, ML. Optical properties of metallic films for vertical-cavity optoelectronic devices. *Appl Opt.* **1998**, *22*, 5271-83.

Transmittance spectra of bare optical disc substrates:

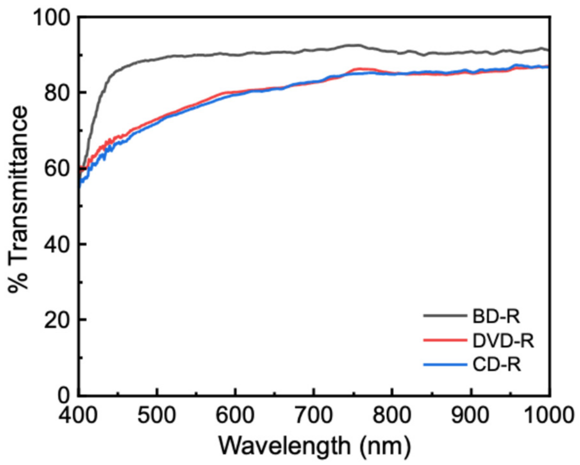

Figure S7. Transmittance spectra of the bare BD-R, DVD-R, and CD-R substrates.
